# New Insights on the miRNA Role in Diabetic Tendinopathy: Adipose-Derived Mesenchymal Stem Cell Conditioned Medium as a Potential Innovative Epigenetic-Based Therapy for Tendon Healing

**DOI:** 10.3390/biom15020264

**Published:** 2025-02-11

**Authors:** Marina Russo, Caterina Claudia Lepre, Gianluca Conza, Nicoletta Tangredi, Giovanbattista D’Amico, Adriano Braile, Antimo Moretti, Umberto Tarantino, Francesca Gimigliano, Michele D’Amico, Maria Consiglia Trotta, Giuseppe Toro

**Affiliations:** 1 Department of Mental, Physical Health and Preventive Medicine, University of Campania “Luigi Vanvitelli”, 80138 Naples, Italy; marina.russo@unicampania.it (M.R.); francesca.gimigliano@unicampania.it (F.G.); 2 School of Pharmacology and Clinical Toxicology, University of Campania “Luigi Vanvitelli”, 80138 Naples, Italy; 3 Department of Experimental Medicine, University of Campania “Luigi Vanvitelli”, 80138 Naples, Italy; caterinaclaudia.lepre@unicampania.it (C.C.L.); nicoletta0@hotmail.it (N.T.); michele.damico@unicampania.it (M.D.); 4 Ph.D. Course in Translational Medicine, University of Campania “Luigi Vanvitelli”, 80138 Naples, Italy; 5 Department of Medical and Surgical Specialties and Dentistry, University of Campania “Luigi Vanvitelli”, 80138 Naples, Italy; gianluca.conza@studenti.unicampania.it (G.C.); antimo.moretti@unicampania.it (A.M.); giuseppe.toro@unicampania.it (G.T.); 6 School of Geriatrics, University of Studies of L’Aquila, 67010 L’Aquila, Italy; giovanbattista.damico.dott@outlook.it; 7 Department of Clinical Sciences and Translational Medicine, University of Rome Tor Vergata, 00133 Rome, Italy; umberto.tarantino@uniroma2.it

**Keywords:** diabetic tendinopathy, microRNAs, adipose-derived mesenchymal stem cell, conditioned medium

## Abstract

Background: Adipose-derived mesenchymal stem cell conditioned medium (ASC-CM) improved the viability and wound closure of human tenocytes (HTCN) exposed to high glucose (HG) by activating the transforming growth factor beta 1 (TGF-β1) pathway. Objectives: Since ASC-CM can also modulate microRNAs (miRNAs) in recipient cells, this study investigated the effects of ASC-CM on the miRNAs regulating tendon repair (miR-29a-3p, miR-210-3p and miR-21-5p) in HG-HTNC. Methods: ASC-CM was obtained by ASCs isolated from the abdominal fat tissue of seven non-diabetic patients. HTNC were cultured in HG for 20 days, then scratched and exposed for 24 h to ASC-CM. qRT-PCR and ELISAs assessed miRNA and target levels. Results: HG-HTNC exhibited a significant downregulation of miRNAs. ASC-CM restored the levels of miRNAs and their related targets involved in tendon repair. Conclusions: The epigenetic modulation observed in HG-HTNC exposed to ASC-CM could be an innovative option in the management of diabetic tendinopathy.

## 1. Introduction

Diabetes is a well-known risk factor for the development of tendinopathies, with diabetic patients showing a higher risk of severe alterations in tendon structure, weakness of mechanical properties and lower healing ability [1,2,3,4,5]. Moreover, both conservative and surgical approaches for diabetic tendinopathy treatment are prone to failure [6,7]. Specifically, corticosteroid injections were less efficient in the treatment of trigger finger in diabetic patients [8], while the incidence of Achilles tendinopathy seems to be increased by physical exercise, generally used as a conservative option, in elderly patients with type 2 diabetes mellitus [9].

To this regard, the injection of adipose-derived mesenchymal stem cells (ASCs) emerged as a novel and interesting therapeutic option, showing good safety, tolerability and efficacy in non-diabetic tendinopathic patients [10,11]. Indeed, following intratendineous injections of ASCs (1 × 10^6^–1 × 10^9^) in patients diagnosed by magnetic resonance imaging (MRI) or ultrasonography (US) with Achilles tendon, rotator cuff disease and elbow and patellar tendinopathy, a marked pain recovery and the amelioration of tendon structure was recorded. Particularly, an improvement of the Visual Analogue Scale (VAS), used to measure pain intensity, was recorded from the second week after treatment and lasted up to 30 months. Also, MRI/US investigations showed significant improvements at intermediate time points and at the end of follow up [12,13,14,15,16,17,18].

However, ASC injections may cause immune responses or decreased treatment satisfaction in the management of tendon disorders [19,20]. To overcome this limitation, the application of ASC conditioned medium (CM) has been explored in preclinical models of non-diabetic tendinopathy [21]. Indeed, intratendineous injections of exosomes contained within ASC-CM improved tendon healing in a rabbit rotator cuff model [22], while microvescicles from ASC-CM promoted suspensory ligament healing in a horse model of tendon damage [23]. In this field, we have previously reported a beneficial role of ASC-CM on tenocytes from a healthy human patellar tendon (HTNC) exposed to high glucose (HG), reporting increased cell viability and wound closure, paralleled by an activation of transforming growth factor beta 1 (TGF-β1) due to specific CM mediators (latent TGF-β1 and thrombospondin 1) [24].

It has been recently reported that ASC-CM is also able to modulate in the recipient cells the expression of microRNAs (miRNAs), short non-coding RNAs able to modify gene expression by epigenetic regulation [25]. To this regard, it is widely accepted that miRNAs are involved in both tendon injury and repair processes by regulating tendon cell differentiation, inflammation, angiogenesis, apoptosis and ECM remodeling [26,27,28,29,30,31]. Interestingly, although a prominent role has been described for miR-29a, miR-210-3p and miR-21-5p as epigenetic modulators of tendon healing and regeneration [26], their role in diabetic tendinopathy and their possible modulation by ASC-CM has been not elucidated yet.

Therefore, in the present study we investigated a possible dysregulation of miR-29a-3p, miR-210-3p and miR-21-5p, and their related targets (collagen type III—Col III; Smad7; vascular endothelial growth factor—VEGF; fibroblast growth factor 2—FGF2) in HTNC exposed to high glucose. Then, we verified if the changes detected in miRNAs and their related targets could be associated with ASC-CM exposure.

## 2. Materials and Methods

### 2.1. Human Subcutaneous Adipose Tissue Collection and Processing

**Figure 1:**
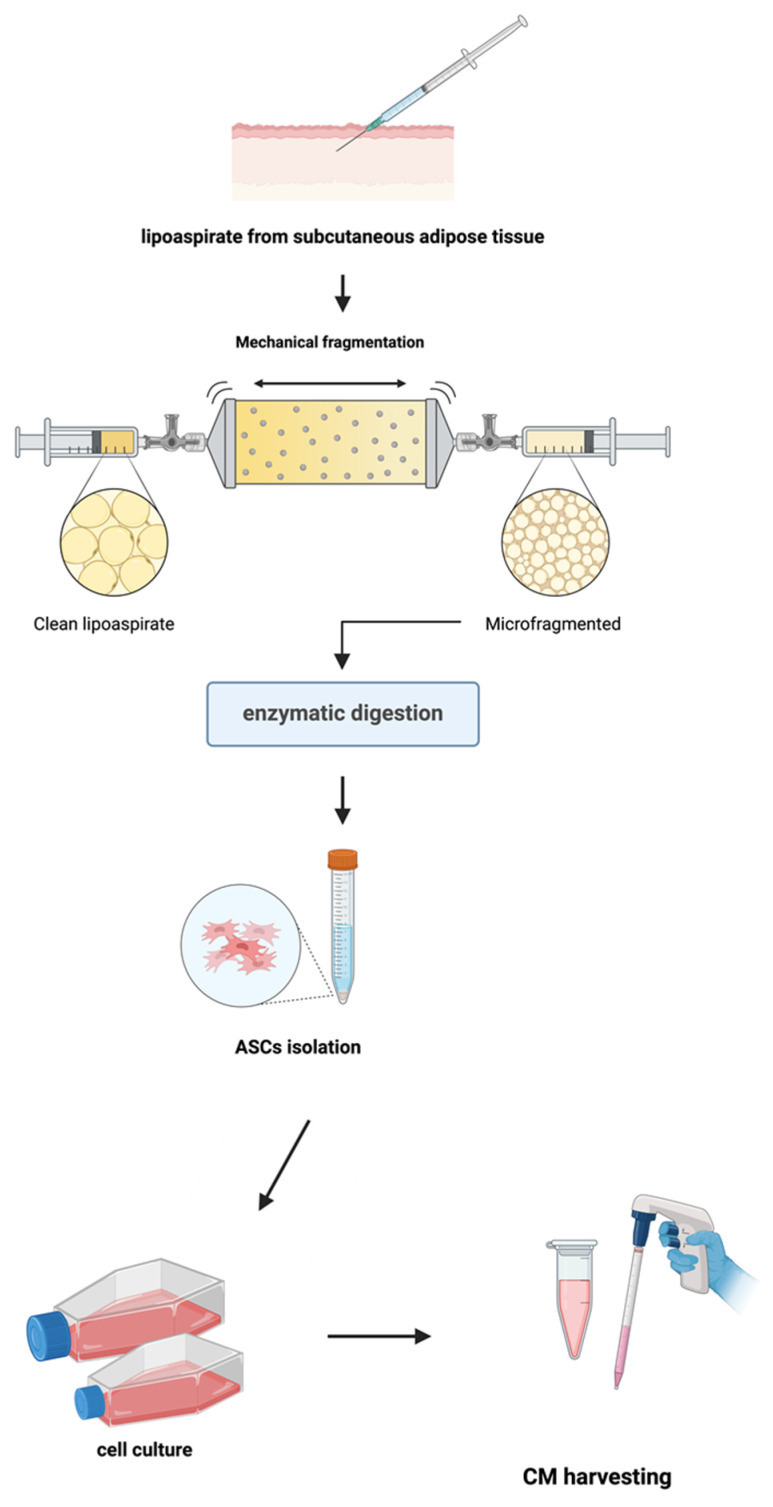
Procedures for ASC isolation and conditioned medium (CM) harvesting.

The collection of lipoaspirated microfragmented adipose tissue (µFAT), and the subsequent in vitro procedures for ASC isolation and culture (Figure 1) adhered to the Declaration of Helsinki and Good Clinical Practice guidelines. Moreover, they were approved by the Ethics Committee of the AOU University of Campania “Luigi Vanvitelli” (protocol number 0035781/i, 15 December 2021).

Subcutaneous adipose tissues were collected at the Unit of Orthopaedics, University of Campania “Luigi Vanvitelli” (Naples, Italy), from patients with early osteoarthritis undergoing abdominal lipoaspiration before hip or knee ASC injections. All the patients participating in the study signed a written informed consent form.

The eligibility criteria determining the inclusion of the subjects into the study were as follows: (I) a minimum age of 18 years; (II) a diagnosis of unilateral/bilateral hip or knee osteoarthritis, confirmed by radiography; (III) joint pain refractory to conservative therapy. Patients exhibiting a diagnosis of diabetes, congenital joint anomalies, joint trauma within 3 months, previous knee or hip prosthetic treatment, joint infiltration within 12 months and a body mass index lower than 18 kg/m^2^ were excluded.

From each donor patient, a 50 mL lipoaspirate sample was collected and microfragmented by using the Lipogems^®^ device (Lipogems International S.p.A.; Milan, Italy), as previously reported [32,33]. From this, a 10 mL µFAT was obtained and used for a single autologous intra-articular injection. Following this procedure, the exceeding µFAT (if any) was used to isolate human ASCs.

### 2.2. Isolation and Characterization of ASCs

The 3 mL µFAT sample was digested with collagenase type II (1 mg/mL, C2-BIOC, Merck; Milan, Italy) in 7 mL of α-Minimum Essential Medium (αMEM; M4526, Merck; Milan, Italy) with 1% of Penicillin–Streptomycin (P/S; AU-L0022, Aurogene; Rome, Italy), 1% of L-Glutamine (L-Glu; 25030081, Thermo Fisher Scientific; Milan, Italy) and 5 mM of glucose, by incubation for 30 min at 37 °C with agitation (200 rpm). Then, cell strainers (70 μm mesh) were used to filter the µFAT sample, which was subsequently suspended in αMEM (1% P/S, 1% L-Glu, 5 mM glucose and 10% Fetal Bovine Serum—FBS; AU-S181H, Aurogene; Rome, Italy) and centrifuged at room temperature for 5 min. The resulting ASC pellet was washed three times with Phosphate Buffer Saline (PBS; 14200, Thermo Fisher Scientific; Milan, Italy) and grown in αMEM (1% P/S, 1% L-Glu, 5 mM glucose and 10% FBS) at 37 °C with 5% CO_2_. For a 10-day period after ASC isolation, the morphology was observed by optical microscopy and the cell viability was assessed by using 3-(4,5-dimethylthiazol-52-yl)-2,5-diphenyltetrazolium bromide (MTT). ASC characterization was performed by immunofluorescence, assessing the presence of CD73, CD90 and CD105 surface antigens, along with the absence of CD34, CD45 and Human Leukocyte antigen–antigen D related (HLA-DR) hematopoietic markers [34,35,36].

### 2.3. ASC-CM Preparation

For the collection of ASC-CM, 4 × 10^5^ ASCs were seeded in culture flasks and cultured in αMEM (1% P/S, 1% L-Glu, 5 mM glucose and 10% FBS). After reaching an 80% confluence, the ASCs were serum starved for 24 h before collecting ASC-CM. This was centrifuged at 200× *g* for 10 min, then sterilized with a 0.22 μm syringe filter.

### 2.4. Purification of ASC-CM miRNAs and Quantitative Reverse Transcription Polymerase Chain Reaction (RT-qPCR)

ExoRNeasy Serum/Plasma Maxi Kit (77064, Qiagen; Milan, Italy) was used to isolate the miRNAs present in ASC-CM using miRNeasy Serum/Plasma Spike-In Control (219610, Qiagen; Milan, Italy) as a positive miRNA external control.

Mature miRNAs were converted to cDNA by using miScript II RT Kit (218161, Qiagen; Milan, Italy), following the manufacturer’s protocol “Reverse Transcription for Quantitative, Real-Time PCR” (first incubation of 60 min at 37 °C, second incubation of 5 min at 95 °C to inactivate the miScript Reverse Transcriptase Mix). Specifically, the miScript Hispec Buffer was used to obtain the sole detection of mature miRNAs by avoiding precursor miRNAs, mRNA and other non-coding RNAs. Then, hsa-miR-29a-3p, hsa-miR-210-3p, hsa-miR-21-5p and Syn-cel-miR-39-3p were amplified by using miScript SYBR^®^ Green PCR Kit (218073, Qiagen; Milan, Italy) and specific miScript Primer Assays (MS00003262, MS00003801, MS00009079 and MS00019789, Qiagen; Milan, Italy). Reactions were carried out in triplicate on a CFX96 Touch TM Real-Time PCR Detection System (Biorad Laboratories Srl, Milan, Italy), according to the manufacturer’s protocol “Real-Time PCR for Detection of Mature miRNA”, by setting the following cycling conditions: 15 min at 95 °C as the initial activation step of HotStarTaq DNA Polymerase; a three-step cycling of 15 s at 94 °C for denaturation, 30 s at 55 °C for annealing and 30 s at 70 °C for extension; followed by fluorescence data collection (repeated for 40 cycles). Data analysis was performed using the 2^−ΔΔCt^ method of relative quantization.

### 2.5. In Vitro Model of Diabetic Tendinopathy

Human tenocytes from healthy human patellar tendons (HTNC) (P10968, Innoprot; Derio, Spain) were used to obtain a cellular model of diabetic tendinopathy. These were cultured in T-75 flasks precoated with poly-l-lysine (2 µg/cm^2^; PLL, Innoprot; Derio, Spain) at 37 °C and 5% CO_2_ in Tenocyte Medium (TCM; P60177, Innoprot; Derio, Spain) containing 1% P/S, 1% Tenocyte Growth supplement and 5% FBS (P60177, Innoprot; Derio, Spain).

HTNC were exposed to normal (5 mM, NG) or high glucose (25 mM, HG) for 20 days by using 20 mM mannitol as positive osmotic control. For the last 24 h (day 21), the NG or HG media were replaced by ASC-CM by using free αMEM as a control in NG or HG cells [24].

Cell morphology was observed daily by optical microscope, while cell viability was determined by MTT assay by seeding 5 × 10^3^ HTCN in PLL (2 µg/cm^2^)-pre-coated 96-well plates after 21 days of treatment. The HTNC viability was reported as % viability = (mean OD treatment/mean OD control) × 100.

For the HTNC scratch assay, 8 × 10^3^ cells/well were seeded in PLL (2 µg/cm^2^)-pre-coated 6-well plates. NG or HG cells were vertically scratched by a 200 μL sterile pipette tip (T0) after 20 days. Then, NG or HG media were replaced by ASC-CM or free αMEM serum as a control for the last 24 h (T24). At T0 and T24, wound closure was observed by Leica DMi1 microscope and was then measured by Image J software 1.47 to assess the % of the initial wound area covered by cells over the 24 h. The wound area at T0 was used as control.

For each assay, triplicates of three independent experiments were performed (N = 9).

### 2.6. Enzyme-Linked Immunosorbent Assays (ELISAs) for miRNA Targets

For HTNC ELISAs, 1 × 10^5^ cells were seeded in PLL (2 µg/cm^2^)-pre-coated T-25 culture flasks. After 21 days of treatment, the cells were trypsinized and centrifuged at 1000 rpm × 5 min to separate the HTNC medium from the HTNC cell pellet. This was washed two times with PBS before assessing the levels of collagen III (Col III), vascular endothelial growth factor (VEGF) and fibroblast growth factor-2 (FGF2), which were assessed in HTNC by ELISAs using commercially available kits (MBS2023209, MyBiosource; San Diego, CA, USA; EH0327 and EH0541 FineTest; Wuhan, China) according to the manufacturer’s protocols.

### 2.7. HTNC qRT-PCR for miRNA Targets

Total RNA was isolated from the HTNC lysates following the miRNeasy Mini kit (217004, Qiagen; Milan, Italy). The NanoDrop 2000c Spectrophotometer (Thermo Fisher Scientific, Waltham, MA, USA) was used to assess RNA concentration and quality. The removal of genomic DNA contaminations was obtained by adding gDNA Wipeout Buffer (205311, Qiagen; Milan, Italy) and RNAse-free water to template RNA and incubating at 42 °C for 2 min according to the manufacturer’s protocol “Reverse Transcription with Elimination of Genomic DNA for Quantitative, Real-Time PCR” (Qiagen; Milan, Italy). Then, the reverse transcription (RT) phase was performed by using Gene AMP PCR System 9700 (Applied Biosystems, Waltham, MA, USA) and the QuantiTect Reverse Transcription kit (205311, Qiagen; Milan, Italy) according to manufacturer’s protocol (first incubation of 15 min at 42 °C, followed by a second incubation of 3 min at 95 °C)

The real-time PCR (qPCR) phase was performed by using the QuantiTect SYBR Green PCR Kit (204143, Qiagen; Milan Italy), together with specific QuantiTect Primer Assays (249900, Qiagen; Milan, Italy) for *Smad7* (QT02397563, Qiagen; Milan, Italy) and Glyceraldehyde 3-phosphate dehydrogenase (*GAPDH*) (QT00079247), as a housekeeping control gene. According to the “Two-Step RT-PCR Standard Protocol”, the triplicate qPCR reactions were carried out on the CFX96 Real-time System C1000 Touch Thermal Cycler (Biorad, Milan, Italy), by setting the following cycling conditions: 15 min at 95 °C as the PCR initial activation step; a three-step cycling of 15 s at 94 °C for denaturation, 30 s at 55 °C for annealing and 30 s at 72 °C for extension; followed by fluorescence data collection (repeated for 40 cycles). The 2^−ΔΔCt^ method was used for the relative quantization of gene expression.

### 2.8. Statistical Analysis

Data were obtained from the triplicates of three independent experiments (N = 9) and reported as mean ± standard deviation (SD). The statistical analysis was performed by using two-way repeated measures Analysis of Variance (ANOVA) followed by post hoc Bonferroni for multiple comparisons (GraphPad Prism 6.0 software—La Jolla, CA, USA). To assess the strength of the association between two parameters, the following tests were performed: Pearson correlation analysis, reporting Pearson’s correlation coefficient (r) values; Kendall correlation, providing the respective Kendall’s tau (τ) values and Spearman’s correlation, reporting Spearman’s correlation coefficient, (ρ). For all the statistical analyses, a *p* value (*p*) < 0.05 was considered statistically significant.

## 3. Results

### 3.1. ASC Isolation and Characterization

µFAT was collected by seven adult non-diabetic female donors (49–59 years), exhibiting joint pain refractory to conservative therapy and diagnosed with unilateral hip osteoarthritis (2), or unilateral (2) or bilateral knee osteoarthritis (3).

ASCs showed a normal morphology and cell viability, with a positive immunoreactivity to CD73, CD90 and CD105, and a negative immunoreactivity to CD34, CD45 and HLA-DR [24].

### 3.2. Detection of miRNAs and Their Targets in HG-HTCN

**Figure 2:**
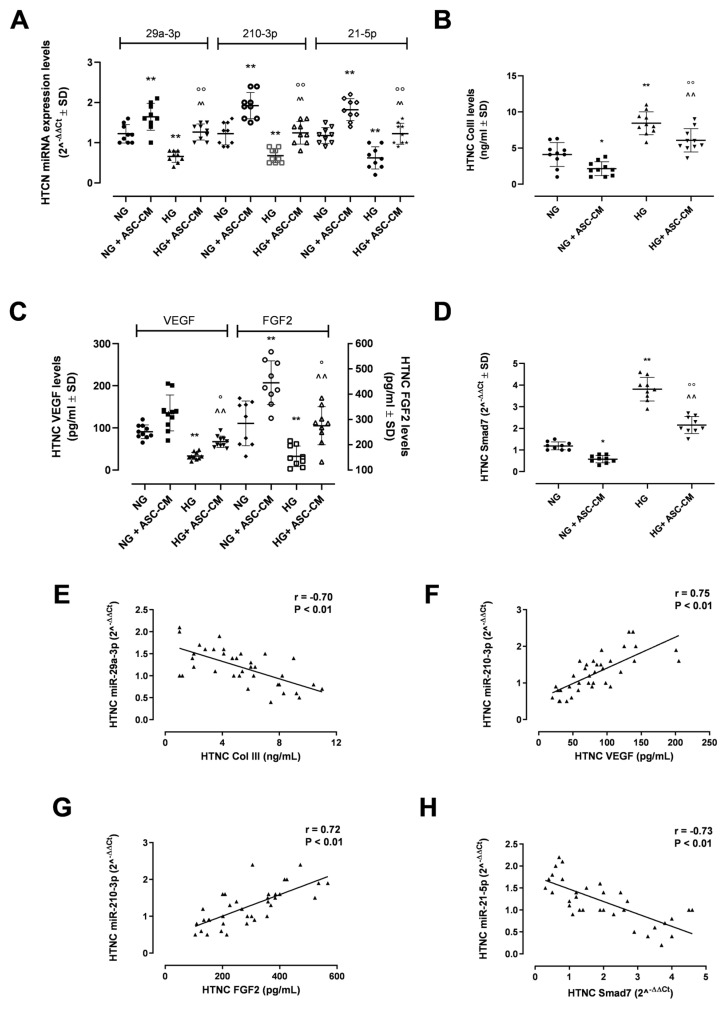
miRNAs and related targets in HG HTNC exposed to ASC-CM. (**A**) miR-29a-3p, miR-210-3p and miR-21-5p expression (2^−ΔΔCt^ ± SD) determined by qRT-PCR in HTCN cultured in normal glucose (NG, 5 mM) or high glucose (HG, 25 mM) for 20 days, then exposed for the last 24 h to free αMEM or ASC-CM (NG+ASC-CM and HG-ASC-CM, respectively); (**B**) Col III (ng/mL ± SD) assessed by ELISA in HTNC; (**C**) VEGF (pg/mL ± SD) and FGF2 (pg/mL ± SD) assessed by ELISA in HTCN; (**D**) *Smad7* mRNA expression (2^−ΔΔCt^ ± SD) determined by qRT-PCR in HTNC; (**E**) Pearson correlation between Col III and miR-29a-3p (r = −0.70, *p* < 0.01); (**F**) Pearson correlation between VEGF and miR-210-3p (r = 0.75, *p* <0.01); (**G**) Pearson correlation between FGF2 and miR-210-3p (r = 0.72, *p* <0.01); (**H**) Pearson correlation between Smad7 and miR-21-5p (r = −0.73, *p* < 0.01); * *p* < 0.05 and ** *p* < 0.01 vs. NG; ° *p* < 0.05 and °° *p* < 0.01 vs. HG; and ^^ *p* < 0.01 vs. NG+ASC-CM.

Starting from the significantly reduced cell viability and altered morphology evidenced by HTCN exposed to 20 days of high glucose followed by 24 h of free αMEM (HG) [24], we aimed, here, to analyze a possible dysregulation of tendinopathy-related miRNAs in HG HTCN. Specifically, a qRT-PCR was performed to assess miR-29a-3p, miR-210-3p and miR-21-5p in the HTNC pellet. The data obtained evidenced a significant downregulation of miR-29a-3p, miR-210-3p and miR-21-5p levels in HG-HTNC compared to cells cultured in normal glucose (NG). Particularly, the decrease observed for miR-29a-3p was −1.72-fold (*p*< 0.05 vs. NG; Figure 2A), with a similar reduction observed for miR-210-3p (−1.78-fold, *p* < 0.05 vs. NG; Figure 2A) and miR-21-5p (−1.40-fold, *p* < 0.05 vs. NG; Figure 2A).

The analysis of miRNA targets (Col III for miR-29a-3p; VEGF and FGF2 for miR-210-3p; Smad7 for miR-21-5p), assessed in the HTNC pellet by ELISA or qRT-PCR, evidenced an upregulation of Col III levels (+2.05-fold, *p* < 0.01 vs. NG; Figure 2B) in HG HTNC, along with a significant downregulation of VEGF (−2.12-fold, *p* < 0.01 vs. NG; Figure 2C) and FGF2 levels (−1.61-fold, *p* < 0.05 vs. NG; Figure 2C). Finally, a dysregulated expression of *Smad7* was evident in HG HTNC, with a marked increase (+2.86-fold, *p* < 0.01 vs. NG; Figure 2D) compared to NG group.

### 3.3. ASC-CM Modulates miRNAs and Their Related Targets

A possible modulation of miRNAs and their related targets in HTNC exposed to ASC-CM was assessed by qRT-PCR or ELISA in the HTNC pellet and was verified by a Pearson correlation analysis. ASC-CM significantly increased miRNA levels in both NG (NG+ASC-CM; miR-29a-3p: +1.34-fold, miR-210-3p: +1.57-fold, miR-21-5p: +1.55-fold; all *p* < 0.05 vs. NG) and HG-HTNC (HG+ASC-CM; miR-29a-3p: +1.78-fold, miR-210-3p: +1.95-fold, miR-21-5p: +1.80-fold; all *p* < 0.05 vs. HG) (Figure 2A). In line with this trend, ASC-CM significantly reduced Col III levels (−1.75-fold, *p* < 0.01 vs. HG) (Figure 2B), which were inversely correlated with miR-29a-3p levels (r = −0.70, *p* < 0.1) (Figure 2E). Moreover, a significant upregulation of VEGF (+1.78-fold, *p* < 0.01 vs. HG) and FGF2 (+1.63-fold, *p* < 0.05 vs. HG) was detected in the HG+ASC-CM group (Figure 2C), with both factors positively correlated with miR-210-3p (VEGF: r = 0.74 and FGF2: r = 0.73, both *p* < 0.01) (Figure 2F,G). Lastly, ASC-CM reduced the *Smad7* expression in HG-HTNC (−1.72-fold, *p* < 0.01 vs. HG) (Figure 2D), expressing a significant negative correlation with miR-21-5p (r = −0.73, *p* < 0.01) (Figure 2H). The significant correlations between miRNAs and their targets were also confirmed by Kendall and Spearman correlation analyses (Table 1).

### 3.4. Correlation Between miRNAs with Wound Closure in HTNC

**Figure 3:**
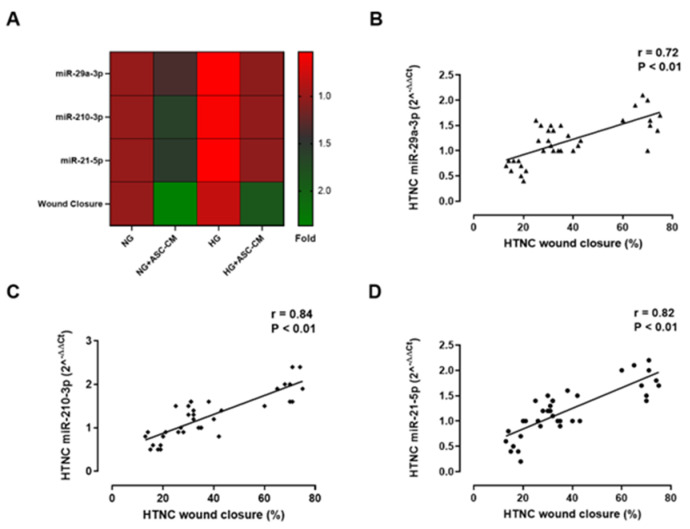
Association between miRNA levels and wound closure in HTNC. (**A**) Heat map of differentially expressed miRNAs (miR-29a-3p, miR-210-3p and miR-21-5p) and wound closure in HTCN cultured in normal glucose (NG, 5 mM) or high glucose (HG, 25 mM) for 20 days, then exposed for the last 24 h to free αMEM or ASC-CM (NG+ASC-CM and HG-ASC-CM, respectively); (**B**) Pearson’s correlation analysis between HTNC wound closure (%) and miR-29a-3p (r = 0.72, *p* < 0.01) and (**C**) miR-210-3p (r = 0.84, *p* < 0.01) and (**D**) miR-21-5p (r = 0.82, *p* < 0.01) levels (2^−ΔΔCt^). r: Pearson’s coefficient.

To verify the possible involvement of miRNAs in HTNC recovery from a scratch assay, which was significantly improved by ASC-CM exposure in HG-HTNC [24], the degree of both miRNA levels and HTNC wound closure were calculated and a Pearson correlation analysis was carried out. The amelioration of wound closure in HG+ASC-CM HTNC was paralleled by higher miRNA levels (Figure 3A). Particularly, a significantly positive association was found between wound closure (% ± SD) with all three miRNAs analyzed here (r = 0.80 for miR-210-3p, *p* < 0.01; r = 0.71 for both miR-29a-3p and miR-21-5p, *p* < 0.01) (Figure 3B, 3C and 3D, respectively).

These results were in line with the data obtained from the Kendall and Spearman correlations analyses (Table 2).

## 4. Discussion

Diabetes is a serious disease with a widespread incidence, with the global adult prevalence estimated to be 10.5% (536.6 million people) in 2021, and rising to 12.2% (783.2 million) in 2045 [37]. It is also considered a risk factor for muscle–skeletal chronic pathologies, promoting an impairment in joint mobility, and increasing the risk of tendon-related pathologies such as Achilles tendon and rotator cuff tendinitis [1,38,39,40,41]. Indeed, marked alterations of the tendon structure have been preclinically and clinically associated with prolonged hyperglycemia, with a detrimental impact on both tendon health and the healing processes [3,4,42]. In a rat model of a rotator cuff tendon associated with persistent type II diabetes, tendons were characterized by the increased expression of tenascin C (TNC) and fatty acid binding protein 4 (FABP4), overall contributing to a significant biomechanical decline of the rotator cuff tendon [42]. Similarly, an RNA sequencing analysis aimed to detect putative changes of gene expression in rotator cuff tendon tissues from diabetic and non-diabetic patients, which evidenced a differential expression of multiple genes mainly involved in the regulation of inflammatory and apoptotic processes, contributing to the progression of rotator cuff tears in diabetic patients and reducing their successful recovery after arthroscopic repair [43,44].

It is well known that the underlying epigenetic mechanisms are strongly dysregulated by specific risk factors such as hyperglycemia, oxidative stress, inflammation and growth factors [45,46]. To this regard, a role for several miRNAs has been illustrated in diabetic retinopathy, nephropathy, cardiomyopathy and neuropathy [47,48,49]. These have been associated with the progression of these diabetic complications or with protection against them [46]. However, to our knowledge, the role of tendon-related miRNAs during diabetic tendinopathy has not been fully elucidated yet. Indeed, to our knowledge, only one manuscript has recently analyzed the involvement of long non-coding RNAs and their mRNA targets in tendon alterations in diabetic patients, without exploring a possible differential expression in the diabetic tendinopathy of tendon-related miRNAs [43].

To date, it is widely accepted that miRNAs are involved in both tendon injury and repair processes by regulating tendon cell differentiation, inflammation, angiogenesis, apoptosis and extracellular matrix (ECM) remodeling. Among the miRNAs associated with tendon healing, a prominent role has been described for miR-29a, miR-210-3p and miR-21-5p, which are upregulated during tendon recovery after injury [31].

Interestingly, although a dysregulation of miR-29a-3p, miR-210-3p and miR-21-5p expression has already been detected in diabetic conditions [50], we here report for the first time that these three miRNAs are significantly downregulated in human tenocytes exposed to prolonged hyperglycemia. Due to their role in promoting tendon healing, this evidence can support the importance of new therapeutic strategies which aim to increase the expression levels of miR-29a-3p, miR-210-3p and miR-21-5p in diabetic tendons to improve their function and healing.

To this regard, several innovative approaches have been developed to obtain effective adjuvant transport systems aimed at increasing miRNA delivery in non-diabetic tendon injuries. Indeed, to favor miR-29a protective effects in tendon disorders, the synthesis of miR-29a oligonucleotide mimetics [51] or precursors [52] have been patented and clinically tested. Indeed, a Phase-I Clinical Trial which aimed to assess the safety, tolerability and pharmacokinetics of a chemically synthesized miR-29a mimic injected in patients with lateral epicondylitis was completed in 2021 [53], with the Phase-II Clinical Study not yet recruiting patients [54]. Other innovative miR-29a delivery systems, such as Adeno-Associated Virus vectors [55] or lipid nanoparticles, have been developed, with the latter tested in a preclinical model of Achilles tendon injury [56]. Similarly, the local injection of synthetic miRNA-210 into injured Achilles tendons in rats accelerated their healing [57].

Recently, the exposure of different cell lines to ASC-CM led to a differential expression of several miRNAs and related mRNA/protein targets in the recipient cells [58]. Also, in our experimental setting, the levels of miR-29a-3p, miR-21-5p and miR-210-3p were found to be upregulated in human tenocytes exposed to ASC-CM both in normal and high glucose conditions. This could be due to the high content of non-coding RNAs exhibited by ASC-CM [59], with miRNAs representing approximately the 44% of non-coding RNAs released in ASC-CM [59]. These exert positive effects in preclinical models of musculoskeletal disorders such as osteoarthritis, osteoporosis, bone defects and cartilage-related pathologies [11,60,61,62,63,64,65,66,67,68,69], along with anti-inflammatory action in tendons, through the induction of M2 macrophage polarization and the inhibition of fatty infiltration [22,70,71,72]. To this regard, we confirm the presence of miR-29a-3p and miR-21-5p in ASC-CM, accordingly with previous evidence [25,62,73], while we also report here for the first time the detection of miR-210-3p, previously found only in BMSC-CM [74]. However, although we cannot assume here that the modulation of the three miRNAs is a direct consequence of the ASC-CM exposure, the reduced miRNA expression found in ASC-CM collected after the 24 h HTNC exposure may suggest an internalization of the three miRNAs in HTNC (Figure S1). This evidence needs to be supported by further experiments aimed at isolating the specific miRNAs from ASC-CM and labelling their internalization in HTNC.

The specific upregulation of these miRNAs was significantly correlated in HTNC with the modulation of the predicted miRNA targets, whose expression was found to be dysregulated in hyperglycemic conditions. Particularly, the exposure of tenocytes, grown in high glucose, to ASC-CM was paralleled by a significant decrease in Col III levels, a main contributor to ECM disorganization and the alteration of tendon structure [75] which inversely correlated with miR-29a-3p levels. Also, Smad7, a stimulator of tendon proliferation, migration and fibrotic activity [76], was reduced in tenocytes exposed to high glucose, and ASC-CM and was negatively associated with miR-21-5p levels. ASC-CM exposure was paralleled by a significant increase in both VEGF and FGF2, two mediators respectively involved in the promotion of angiogenesis and vascular permeability and in the promotion of tendon-derived stem cells during tendon healing [77]. These positively correlated with HTNC miR-210-3p levels.

Overall, our results suggest the potential use of ASC-CM as an innovative epigenetic modulator in diabetic tenocytes. However, the study presents some limitations regarding the effective role of miRNAs in modulating their related targets in HG HTCN. Indeed, transfection of HG HTNC with miRNA mimics or inhibitors in place of ASC-CM exposure, followed by measurements of changes in Col III, VEGF, FGF2 and SMAD7, could directly demonstrate the correlation data presented between miRNAs and their target by strengthening the evidence presented here. Furthermore, the identification of additional miRNAs and their related mediators in ASC-CM could highlight new molecular mechanisms underlying the positive effects of ASC-CM in diabetic tendon healing.

## 5. Conclusions

Although further in vitro and in vivo studies are needed to evaluate ASC-CM as a miRNA-containing medium capable of modulating, in vitro, the activity of diabetic tenocytes, the current results pave the way to an ASC-CM-mediated promotion of the epigenetic mechanisms that could be exploited as new approaches to the management of diabetic tendinopathy.

## Figures and Tables

**Table 1:** Kendall and Spearman correlation analyses between miRNAs and related targets. τ: Kendall’s tau values; and ρ: Spearman’s correlation coefficient.

	Col III	VEGF	FGF2	Smad7
miR-29a-3p	τ: −0.50; ρ: −0.66 *p* < 0.001	-	-	-
miR-210-3p	-	τ: 0.62; ρ: −0.81 *p* < 0.001	τ: 0.58; ρ: −0.75 *p* < 0.001	-
miR-21-5p	-	-	-	τ: −0.55; ρ: −0.76 *p* < 0.001

**Table 2:** Kendall and Spearman correlation analyses between miRNAs and HTNC wound closure. τ: Kendall’s tau values; and ρ: Spearman’s correlation coefficient.

	Wound Closure
miR-29a-3p	τ: 0.50; ρ: 0.70; *p* < 0.001
miR-210-3p	τ: 0.64; ρ: 0.81; *p* < 0.001
miR-21-5p	τ: 0.61; ρ: 0.80; *p* < 0.001

## Data Availability

All data are included within the article.

## Supplementary Materials

The following supporting information can be downloaded at: https://www.mdpi.com/article/10.3390/biom15020264/s1, Figure S1: Determination of miRNAs in ASC-CM.
