# Supplementary material for: New Insights on the miRNA Role in Diabetic Tendinopathy: Adipose-Derived Mesenchymal Stem Cell Conditioned Medium as a Potential Innovative Epigenetic-Based Therapy for Tendon Healing
Source: Biomolecules. 2025 Feb 11;15(2):264. doi: 10.3390/biom15020264 (PMC11852990; doi:10.3390/biom15020264)
Supplement: Supplementary file 1 [file biomolecules-15-00264-s001.zip › Suppl_Biomolecules.pdf]

biomolecules

MDPI

Article

New Insights on the miRNA Role in Diabetic Tendinopathy: Adipose-Derived Mesenchymal Stem Cell Conditioned Medium as a Potential Innovative Epigenetic-Based Therapy for Tendon Healing

Marina Russo [1,2,†], Caterina Claudia Lepre [3,4,†], Gianluca Conza [5], Nicoletta Tangredi [3], Giovanbattista D'Amico [6], Adriano Braile [5,7], Antimo Moretti [5], Umberto Tarantino [7], Francesca Gimigliano [1], Michele D'Amico [3], Maria Consiglia Trotta [3,*] and Giuseppe Toro [5]

1 Department of Mental, Physical Health and Preventive Medicine, University of Campania "Luigi Vanvitelli", 80138 Naples, Italy; marina.russo@unicampania.it (M.R.); francesca.gimigliano@unicampania.it (F.G.)
2 School of Pharmacology and Clinical Toxicology, University of Campania "Luigi Vanvitelli", 80138 Naples, Italy
3 Department of Experimental Medicine, University of Campania "Luigi Vanvitelli", 80138 Naples, Italy; caterinaclaudia.lepre@unicampania.it (C.C.L.); nicoletta0@hotmail.it (N.T.); michele.damico@unicampania.it (M.D.)
4 PhD Course in Translational Medicine, University of Campania "Luigi Vanvitelli", 80138 Naples, Italy
5 Department of Medical and Surgical Specialties and Dentistry, University of Campania "Luigi Vanvitelli", 80138 Naples, Italy; gianluca.conza@studenti.unicampania.it (G.C.); antimo.moretti@unicampania.it (A.M.); giuseppe.toro@unicampania.it (G.T.)
6 School of Geriatrics, University of Studies of L'Aquila, 67010 L'Aquila, Italy; giovanbattista.damico.dot@outlook.it
7 Department of Clinical Sciences and Translational Medicine, University of Rome Tor Vergata, 00133 Rome, Italy; umberto.tarantino@uniroma2.it
* Correspondence: mariaconsiglia.trotta2@unicampania.it
† These authors contributed equally to this work.

SUPPLEMENTARY MATERIALS

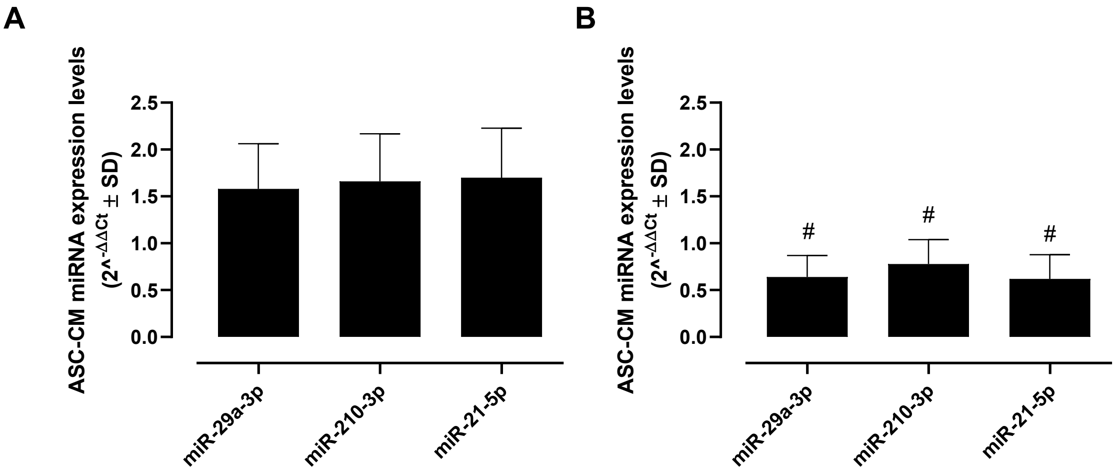

Figure S1. Determination of miRNAs in ASC-CM. miR-29a-3p, miR-210-3p and miR-21-5p levels ($2^{\wedge\Delta\Delta Ct}$ ± SD) before (A) and after (B) HTCN exposure to ASC-CM. ** P < 0.01 vs active TGF-β1; ^^ P < 0.01 vs same group before HTCN exposure.

 https://doi.org/10.3390/biom15020264
